# Editorial: Regenerative biologics for musculoskeletal injuries

**DOI:** 10.3389/fpain.2024.1400548

**Published:** 2024-03-26

**Authors:** Ashim Gupta, Anish G. Potty, Nicola Maffulli

**Affiliations:** ^1^ Future Biologics, Lawrenceville, GA, United States; ^2^ Regenerative Orthopaedics, Noida, India; ^3^ South Texas Orthopaedic Research Institute (STORI Inc.), Laredo, TX, United States; ^4^ Department of Trauma and Orthopaedic Surgery, Faculty of Medicine and Psychology, University La Sapienza, Rome, Italy; ^5^ Centre for Sports and Exercise Medicine, Barts and the London School of Medicine and Dentistry, Mile End Hospital, Queen Mary University of London, London, England; ^6^ School of Pharmacy and Bioengineering, Keele University School of Medicine, Stoke on Trent, United Kingdom

**Keywords:** allogenic platelet-rich plasma, umbilical cord, Wharton’s jelly, amniotic membrane, knee osteoarthritis, hip osteoarthritis, temporomandibular joint, lumbar radiculopathy

**Editorial on the Research Topic** Regenerative biologics for musculoskeletal injuries

Musculoskeletal (MSK) injuries affect bones, tendons, ligaments, muscles and nerves, and, in the long-term, result in pain, diminished function, instability and osteoarthritis (OA) (1, 2). MSK injuries are conventionally managed using non-pharmacological modalities such as immobilization, physiotherapy and activity modification; pharmacological agents such as non-steroidal anti-inflammatory drugs, narcotics, corticosteroids and viscosupplementation; minimally invasive procedures such as radiofrequency ablation; and surgery in advanced stages or when conservative therapies have been unsuccessful (1–3). However, these treatment options have shortcomings and potential side-effects (1–3).

The last decade has seen a significant increase in the use of biologics for MSK regenerative medicine applications. Biologics can be derived from autologous sources, such as platelet-rich plasma (PRP), or allogenic sources, such as perinatal tissue derived formulations (1–3). In our research topic, “*Regenerative Biologics for Musculoskeletal Injuries*”, three articles highlighted the potential of these biologics for different MSK conditions, and one emphasized the importance of ultrasound guidance to improve injection accuracy.

Gupta et al., in a mini review, summarized the outcomes of preclinical and clinical studies using allogenic PRP for the management of knee and hip OA. Three preclinical and one clinical study, and one clinical using allogenic PRP for the treatment of knee and hip OA, respectively, were identified. Administration of allogenic PRP was safe and potentially effective in knee or hip OA patients. However, more studies are warranted to establish the safety and efficacy of allogenic PRP in knee or hip OA patients.

Aratikatla et al., in a mini review, summarized the outcomes of *in vitro*, preclinical and clinical studies using umbilical cord (UC)-derived tissue and mesenchymal stem cells (MSCs) for the management of temporomandibular joint (TMJ) disorders. Two *in vitro*, three preclinical and one clinical study using UC tissue and MSCs were identified. The results showed potential safety and efficacy of UC tissue and MSCs for the management of TMJ ailments. However, more studies are required to support its use in patients suffering with TMJ disorders.

Miedema and Anderson, in a case series analysis of real-world clinical practice, assessed the safety and efficacy of epidural injection of amniotic membrane (AM)/umbilical cord (UC) particulate in 12 lumbar radiculopathy patients, none of whom suffered any adverse effects. Administration of AM/UC particulate resulted in significant reduction in the average pain score at 21.3 ± 11.1 months follow-up compared to the baseline. The results showed preliminary safety and potential applicability of AM/UC particulate epidural injection in patients with lumbar radiculopathy. More studies are needed to further establish the safety and efficacy of AM/UC particulate in patients with lumbar radiculopathy to justify its routine clinical usage.

Ghandour et al., in a retrospective case series, evaluated the effectiveness of office-based portable ultrasound guided intra-articular injections in patients with different pathologies of the fore- and mid-foot. 16 patients were enrolled and were administered 2 ml of 1% lidocaine and 12 ml of 40 mg/ml Kenalog. No adverse events were reported. Significant reduction in the pain scores at 3 months follow-up compared to the baseline were observed. This can be attributed to better injection accuracy achieved by the ultrasound guidance. More studies are required to determine the effectiveness of portable ultrasound compared to the conventional ultrasound guided and non-guided injections to assess its true potential and justify routine clinical use for treating foot pathologies.

In addition to the need for more studies, including non-randomized and randomized controlled trials (RCTs), to further establish the safety and efficacy of aforementioned biologics, future studies assessing the effectiveness of one biologic compared to the another are warranted to aid clinicians in determining the optimal biologic for MSK regenerative medicine applications. Moreover, even though RCTs are considered most trustworthy and the majority of the clinical recommendations are given based on the results obtained from them, the conditions in a RCT are controlled (i.e., study participants are selected with the goal of reducing comorbidities and the study protocol is designed to guarantee highest participant(s) and investigator(s) compliance) and they do not incorporate the divergent characteristics of real-world populations (4, 5). Therefore, it is vital to incorporate real-world evidence with RCTs to complement it and to improve the wholeness of evidence-based medicine evaluations.

In conclusion, biologics and their administration routes are hot topics in musculoskeletal medicine. The topic is popular, but well validated scientific data still need to be produced.
